# Indole Derivatives as Cyclooxygenase Inhibitors: Synthesis, Biological Evaluation and Docking Studies

**DOI:** 10.3390/molecules23061250

**Published:** 2018-05-24

**Authors:** Mashooq Ahmad Bhat, Mohamed A. Al-Omar, Mohammad Raish, Mushtaq Ahmad Ansari, Hatem A. Abuelizz, Ahmed H. Bakheit, Ahmed M. Naglah

**Affiliations:** 1 Department of Pharmaceutical Chemistry, College of Pharmacy, King Saud University, Riyadh 11451, Saudi Arabia; malomar1@ksu.edu.sa (M.A.A.-O.); habuelizz@ksu.edu.sa (H.A.A.); abujazz@gmail.com (A.H.B.); 2 Department of Pharmaceutics, College of Pharmacy, King Saud University, Riyadh 11451, Saudi Arabia; mraish@ksu.edu.sa; 3 Department of Pharmacology, College of Pharmacy, King Saud University, Riyadh 11451, Saudi Arabia; muansari@ksu.edu.sa; 4 Department of Chemistry, Faculty of Science and Technology, El-Neelain University, P.O. Box 12702, Khartoum 11121, Sudan; 5 Department of Pharmaceutical Chemistry, Drug Exploration and Development Chair (DEDC), College of Pharmacy, King Saud University, Riyadh 11451, Saudi Arabia; amnaglah@gmail.com; 6 Peptide Chemistry Department, Chemical Industries Research Division, National Research, Centre, 12622-Dokki, Cairo, Egypt

**Keywords:** indole derivatives, docking, anti-inflammatory activity, analgesic activity, ulcerogenic activity, cyclooxygenase expression

## Abstract

A new series of 2-(5-methoxy-2-methyl-1*H*-indol-3-yl)-*N*′-[(*E*)-(substituted phenyl) methylidene] acetohydrazide derivatives (**S1**–**S18**) were synthesized and evaluated for their anti-inflammatory activity, analgesic activity, ulcerogenic activity, lipid peroxidation, ulcer index and cyclooxygenase expression activities. All the synthesized compounds were in good agreement with spectral and elemental analysis. Three synthesized compounds (**S3**, **S7** and **S14**) have shown significant anti-inflammatory activity as compared to the reference drug indomethacin. Compound **S3** was further tested for ulcerogenic index and cyclooxygenase (COX) expression activity. It was selectively inhibiting COX-2 expression and providing the gastric sparing activity. Docking studies have revealed the potential of these compounds to bind with COX-2 enzyme. Compound **S3** formed a hydrogen bond between OH of Tyr 355 and NH_2_ of Arg 120 with carbonyl group and this hydrogen bond was similar to that formed by indomethacin. This study provides insight for compound **S3**, as a new lead compound as anti-inflammatory agent and selective COX-2 inhibitor.

## 1. Introduction

Inflammation is an important mechanism to defend the body against infection or any physical or chemical offense. This protection mechanism is involved in common life-threatening diseases, including autoimmune diseases such as rheumatoid arthritis, Crohn’s disease or inflammatory bowel syndrome [1]. Prostaglandin is the essential moderator in the inflammation process. Cyclooxygenase enzyme (COX) catalyzes the conversion of arachidonic acid into the prostaglandin E_2_, because of the instability of prostaglandin E_2_, an isomerase enzyme specific to this intermediate, converts it to many prostanoids [2]. Anti-platelet activity and the protection of the gastro-intestinal tract are the beneficial effect of the prostaglandin. Fever and pain associated with the inflammation are the unwanted effects of prostaglandin [3]. Two isoforms of COX have been identified, COX-1 and COX-2. The gastrointestinal (GI) tract cytoprotection effect is provided by COX-1 while the inflammation is mediated by COX-2 [4].

Non-steroidal anti-inflammatory drugs (NSAIDs) are frequently used and are effective analgesics in palliative care. However, use of these medicines is limited, because of adverse side effects. The gastrointestinal toxicity is a common side effect, which is sometimes concomitant with gastro duodenal perforation, bleeding or ulcer complications. This toxicity is caused by the inhibition of cyclooxygenase-1 (COX-1). Highly risked patients with NSAIDs were prescribed with gastro protective drugs, such as the proton pump inhibitors. Therefore, selective inhibitors of the cyclooxygenase-2 (COX-2) is attempted to be developed in order to reduce the side effects. Studies have confirmed the reduction of the gastrointestinal toxicity incidences by the COX selective drugs [5]. Most of the commonly used NSAIDs have high selectivity to COX-1 more than COX-2. Therefore, their use for long time will cause gastric irritation, ulcer or bleeding [6]. The selective inhibition of COX-2 will have the same anti-inflammatory effect as the non-selective inhibitors but with less gastrointestinal adverse effect incidence. However, it allows the cytoprotective prostaglandin synthesis; reduce the bleeding and ulceration [7]. Drugs such as celecoxib, rofecoxib, etoricoxib and valdecoxib have been approved as selective COX-2 inhibitors. These drugs were having fewer gastrointestinal side effects compared to traditional NSAIDs. Long term uses of COX-2 inhibitors have been reported with cardiovascular side effects except celecoxib.

Indomethacin is a NSAID and one of the indole acetic acid derivative, which is known to cause ulcers for its users. However, its safety profile has been improved by chemical modifications [8]. This has shown, that modification by synthesis has high possibility to provide derivatives with significant anti-inflammatory activity and fewer side effects. This will be provided by the COX-2 inhibitors as a solution of the aforementioned criteria [9,10]. Indole is an important scaffold in the field of medicinal chemistry. Indole derivatives have been reported to possess significant pharmacological activities. Derivatives of indole have been used as anti-inflammatory, analgesic and antipyretic agents [11,12,13]. Schiff bases have been reported to possess various pharmacological activities e.g., anti-inflammatory activity [14], anti-tubercular activity [15] and anticonvulsant activity [16,17].

The aim of the present study was to synthesize novel indole derivatives and to evaluate their potential as anti-inflammatory and analgesic agents with gastric sparing activity.

## 2. Results and Discussion

**Scheme 1:**
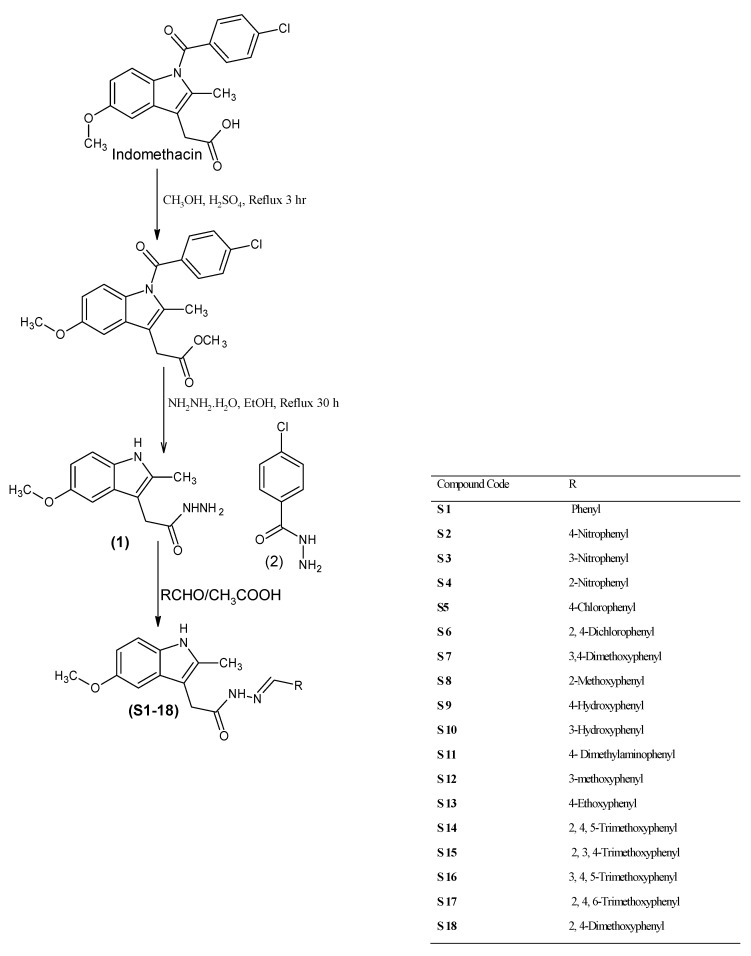
Synthetic route of compounds (**S1**–**S18**).

Indole hydrazide, 2-(5-methoxy-2-methyl-1H-indol-3-yl) acetohydrazide (**1**), was used as a starting material for the synthesis of various substituted indole derivatives [18]. Schiff bases were obtained by refluxing indole hydrazide (**1**) with differently substituted benzaldehydes in ethanol with Glacial acetic acid as catalyst. The synthesis of these compounds was achieved through an efficient synthetic route (Scheme 1). The ^1^H-NMR and ^13^C-NMR was used to assign the structures of synthesized compounds. Moreover, the structures were characterized by elemental analysis, mass spectrometry and FT IR. The structure of the 2-(5-methoxy-2-methyl-1*H*-indol-3-yl) acetohydrazide (**1**) has shown similar NMR splitting pattern and δ-values (δ_H_ & δ_C_) as that of indomethacin, including the pattern of three aromatic protons (H-3, H-5 and H-6) and their ^13^C-signals. The structures of the indole hydrazide derivatives were established on the basis of ^1^H-NMR analysis, which was confirmed by the disappearance of –NH_2_ protons at 4.26 ppm. The detailed results of ^1^H-NMR and ^13^C-NMR and MS are provided in the experimental part. The presence of all carbon atoms for compounds (**S1**–**S18**) were confirmed by ^13^C-NMR spectra. Molecular weights of compounds were confirmed by mass spectra. Molecular ion peak were observed in all the compounds.

### 2.1. Anti-Inflammatory Activity

The synthesized compounds (**S1**–**S18**) were screened by the carrageenan-induced paw edema method for anti-inflammatory activity [19]. The anti-inflammatory activity of the tested compounds after 2 h ranges between 7.35% to 62.69% inhibition. After 3 h, the inhibition was 7.06% to 63.69%, compared to the reference drug indomethacin that showed 77.23% inhibition after 2 h and 76.89% inhibition after 3 h. The compounds (**S3**, **S7** and **S14**) were found to be most potent compounds of the series compared to the reference drug indomethacin (Table 1). A significant anti-inflammatory activity of these compounds was observed because of their hydrazide substitution. However, the substitution of the hydrazide with 3-nitrophenyl as in compound **S3** has shown 61.99% inhibition after 2 h and 61.20% inhibition after 3 h. Whereas, the substitution with 3,4-dimethoxyphenyl as in compound **S7** was observed to have 61.47% and 62.24% inhibition after 2 h and 3 h, respectively. The inhibition by compound **S14** with 2,4,5-trimethoxyphenyl substitution was 62.69% after 2 h and 63.69% after 3 h. These observations provided the base for further testing and development of these compounds. The indole Schiff base derivatives (**S1**–**S18**) having 3-nitrophenyl; 3,4 dimethoxyphenyl and 2,4,5 trimethoxyphenyl substitutions showed the maximum anti-inflammatory activity. Minimum anti-inflammatory activity was shown by the compound containing 2-methoxyphenyl substitution. The compounds containing 2-nitrophenyl; 2,4-dichlorophenyl and 4-dimethylaminophenyl substitution showed more than 50% inhibition.

### 2.2. Analgesic Activity

The compounds that exhibited significant anti-inflammatory activity were selected for the analgesic activity (Table 2). Hot-plate method was used for testing the analgesic activity [20]. The compound **S14** (R = 2,4,5-trimethoxyphenyl) expressed significant analgesic activity of 70.27% inhibition, compared with the reference drug indomethacin with 84.09% inhibition. The analgesic activity of compound **S3** (R = 3-nitrophenyl) was found to be 61.36%. The exchange of the nitro group at 3 position of the substituted phenyl with hydroxyl group has shown analgesic activity of 62.50% in compound **S10**. The compound **S17** (R = 2,4,6-trimethoxyphenyl) was found to have significant analgesic activity with 61.53% inhibition. The aforementioned analgesic results have proven that compounds **S14**, **S10**, **S17**, **S3** and **S9** have a significant analgesic activity.

### 2.3. Ulcerogenic Activity

All the compounds were further evaluated for their ulcerogenic and lipid peroxidation activity (Table 3). The mucosal damage after oral administration of the compounds was examined [21]. Equimolar concentrations of each compound were administered as oral doses to the examined animals. The examined compounds demonstrated a significant ulcerogenic reduction activity ranging between 0 to 0.966 ± 0.16. The maximum ulcerogenic reduction activities were observed in compounds **S4**, **S6**, **S11**, **S15** and **S17**. The reduction of the ulcerogenic activity for compound **S3** (R = 3-nitrophenyl) and **S7** (R = 3,4-dimethoxyphenyl) were 0.116 and 0.432, respectively. However, the ulcerogenic reduction activity of compound **S14** (R = 2,4,5-trimethoxyphenyl) was 0.616. These results of the reduction of the ulcerogenic activity were compared with the standard drug indomethacin (0.948 ± 0.21). It was noticed that the highest activity was observed with the 2-nitrophenyl substitution as in compound **S4** compared with the 3-nitrophenyl as in compound **S3**. The methoxy group substitution on the phenyl presented a significant ulcerogenic reduction. However, the 3,4-dimethoxyphenyl substitution has shown 54% reduction in the ulcerogenic activity in compound **S7**, compared to **S15** with an added third methoxy group at position 2 to be 2,3,4-trimethoxyphenyl substitution. Similar observation was in compound **S17** with (R = 2,4,6-trimethoxyphenyl) that showed 0 ulcerogenic activity. This was compared with compound **S14** with (R = 2,4,5-trimethoxyphenyl) substitution with 35% of the ulcerogenic reduction activity. It has been known that the reduction of malondialdehyde (MDA) content in liver and kidney tissues is concomitant with the reduction of the ulcerogenic activity [22]. The gastric mucosa was scrapped after the ulcerogenic activity screening for lipid peroxidation determination in the gastric mucosa. The reference drug indomethacin has shown highest lipid peroxidation in liver as 8.16 nmoL/100 mg and in kidney as 6.70 nmoL/100 mg. However, compounds with less ulcerogenic activity have also shown reduction in the lipid peroxidation values.

### 2.4. Compound ***S3*** Biological Characterization

Three compounds were found to have a significant anti-inflammatory and analgesic activity. These compounds can be ordered according to their anti-inflammatory and analgesic activity as **S14** > **S3** > **S7**. However, these compounds were also evaluated for their less ulcerogenic effect in comparison to the reference drug indomethacin as **S3** > **S7** > **S14**. Compound **S3** has shown a maximum reduction in the lipid peroxidation and gastric ulceration. Therefore, Compound **S3** with 3-nitrophenyl substitution was found to be most the potent anti-inflammatory and analgesic derivative, as well as, a significant gastric sparing activity.

### 2.5. Gastro-Protective Effect of Compound ***S3***

**Figure 1:**
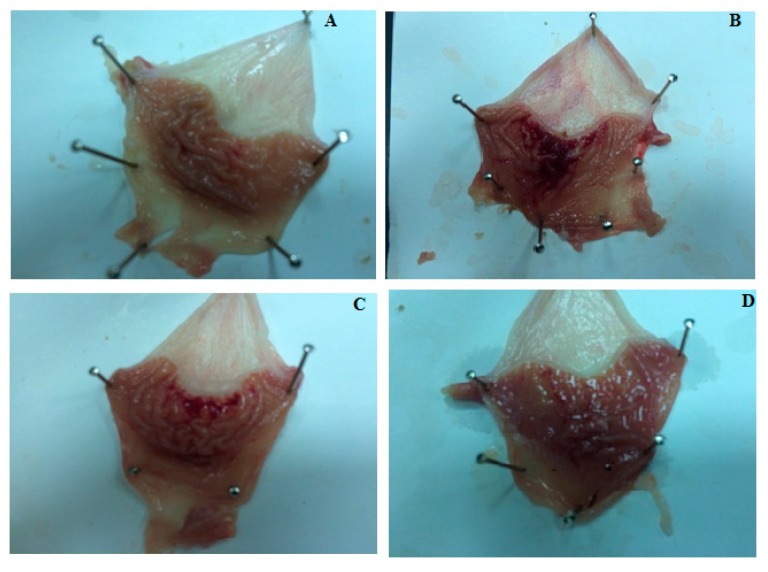
(**A**) Gastric sections of control rats showing normal mucosa. (**B**) Gastric section of untreated rats showing mucosal ulceration. (**C**) Gastric section of compound **S3** treated rats (25.6 mg/kg) showing intact mucosa with mild ulceration (**D**) Gastric section of indomethacin-treated rats (25 mg/kg) showing focal mucosal ulceration.

**Figure 2:**
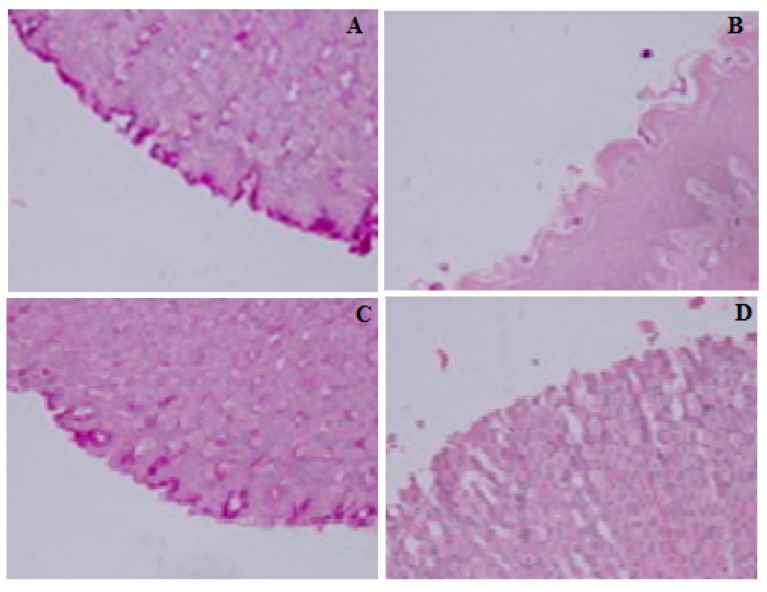
The ethanol-induced gastric ulcers after treatment with (**A**) normal control, (**B**) ethanol, (**C**) compound **S3** (25.6 mg/kg) with ethanol and (**D**) indomethacin (25 mg/kg) with ethanol. PAS staining indicates the mucus secretion in the gastric glands.

**Figure 3:**
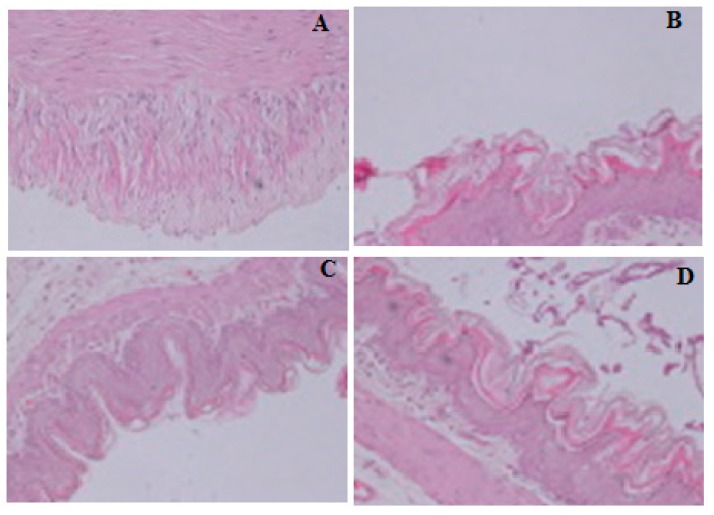
(**A**) Gastric section showing intact gastric mucosa of control rats. (**B**) Gastric section showing gastric mucosal ulceration consisting of necrosis, cellular debris neutrophils and degenerated epithelial cells of ethanol treated rats. (**C**) Gastric section showing focal mucosal ulceration consisting of predominantly neutrophils and degenerated epithelial cells of compound **S3** (25.6 mg/kg) and ethanol treated rats. (**D**) Gastric section showing intense mucosal ulceration consisting of necrosis, cellular debris, neutrophils and degenerated epithelial cells of indomethacin (25 mg/kg) and ethanol treated rats.

The gastro-protective activity was evaluated using indomethacin with ethanol-induced and the ulcer control rat, ethanol-induced rats [23]. It was compared with pre-treated rats using compound **S3**. It was found that 12.34 ulcer index for the indomethacin with ethanol-induced rat and 7.83 for the ethanol-induced rat. However, a significant decrease in the ulcer index of the pre-treated rat with compound **S3** was found to be 2.83 (Table 4). The gastric mucosa was increased in the rats pre-treated with compound **S3**. The gastric mucus has a significant role in the gastric protection. The pre-treatment of the rats with compound **S3**, plays a crucial role by enhancing the free mucus in augmenting the gastro-protection activity in comparison to the ulcer control mucosa. The mucus is adhered to the gastric surface because it is formed from mucin-type glycoprotein. This mucus protects the underneath epithelium against pepsin, acid, necrotizing agents such as, indomethacin and ethanol. Moreover, it is involved in repairing the gastric epithelium damage and defending the mucosa from the aggregation of mechanical factors and chemical [24]. Thus, the gastro-protective effect of compound **S3** against indomethacin and ethanol is demonstrated by improving the mucosal content (Figure 1). The glycogen level of the control and the pre-treated animal was also checked using the Periodic Acid-Schiff (PAS). The ulcers induced by ethanol or indomethacin causes extensive gastric mucosal injury. Moreover, they exhibit hemorrhagic and necrotic lesions which infiltrate into the mucosa and causes edema and leukocyte infiltration. However, the pre-treatment with compound **S3** resulting in expansion of mucus gel layer that with continuous PAS-positive that lines the gastric mucosal surface (Figure 2 and Figure 3). The magenta staining color is exhibited with the compound **S3** pre-treated group. The tissue has a normal glandular pattern and mild leucocyte infiltration (Figure 2C and Figure 3C). On the other hand, the gastric specimen from the control and the indomethacin pre-treatment did not exhibit the magenta staining color. As it can be seen in (Figure 2B and Figure 3B), the ethanol-induced ulcer exhibits pervasive injury to the gastric mucosa. The pre-treatment with indomethacin causes severe ulcer and injury (Figure 2D and Figure 3D). In gastro-protective experiments, the ulcer control rats, ethanol-induced and indomethacin with ethanol-induced rats, revealed severe mucosal damage with an ulcer index of 7.83 ± 0.33 and 12.34 ± 0.73, respectively. The compound **S3** pre-treated rats exhibited a significant decrease in ulcer index (2.83 ± 0.87), and less mucosal damage. These results clearly indicate that compound **S3** has gastro-protective activity. Mucus production by gastric mucosa increased gradually in the experimental rats pre-treated with compound **S3**. Gastric mucus plays crucial role in gastro-protection. The pre-treatment with compound **S3** significantly augmented the gastro-protective activity, with enhancement of the free mucus when compared to the mucus of ulcer control animals. Thus, compound **S3** has gastro-protective activity against ethanol and indomethacin ethanol induced gastric ulcer by improving mucosal content.

### 2.6. Toxicity of Compound ***S3***

Karber method was used to determine the LD_50_ of compound **S3** [25]. A 24 h’s observation was made for the toxicity symptoms and mortality. The dead animals were counted at the end of the study and the LD_100_ was calculated. The LD_50_ of compound **S3** was found to be 35 mg/kg.

### 2.7. COX-1 and COX-2 Protein Expression

**Figure 4:**
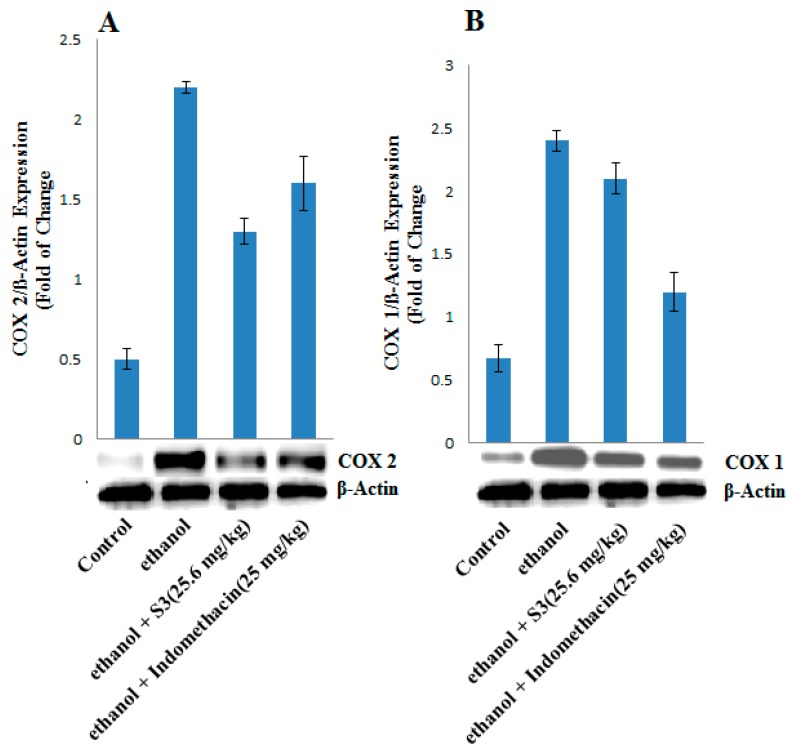
Western blot analysis of COX-2 (**A**), and COX-1 (**B**) expression in the rat gastric mucosa damaged by ethanol. The ethanol-administered rats showed marked increases in COX-2, and COX-1 expression in the gastric mucosa (a representative blot is shown). These increases were significantly suppressed by pre-treatment with compound **S3** (25.6 mg/kg) or indomethacin (25 mg/kg), which were given 1 h before ethanol administration.

A Western blot to assess the protein expression was applied in the pre-treatment with ethanol, indomethacin or compound **S3** [26]. The indomethacin pre-treatment has shown gastro-toxicity because of the potent COX-1 expression inhibition in the ethanol-induced ulcer (Figure 4B). However, compound **S3** has shown potent COX-2 expression inhibition with low COX-1 expression (Figure 4A). This demonstrates that the gastro-protection activity of compound **S3** caused by attenuating the ethanol lesions induces the gastric mucus and decrease the production of COX-2.

### 2.8. Docking Studies of Compounds *(**S1**–**S18**)* to the COX-1/COX-2

**Figure 5:**
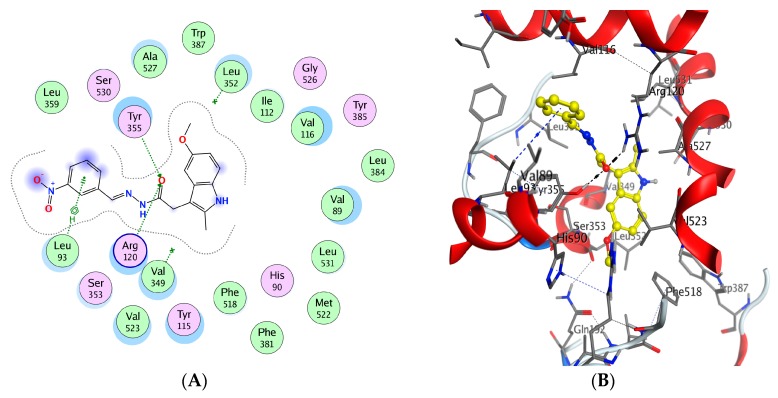
Compound **S3** in the binding site of COX-2 (PDB: 4COX). (**A**,**B**) represents the 2D and 3D docking view of compound **S3** with binding site of COX-2. In 2D, green dashed line represents hydrogen bonding; green dashed line represents pi-H bonding; residues which are colored light green, represents hydrophobic interactions. In 3D, black dashed line represents hydrogen bonding; blue dashed line represents pi-H bonding.

**Figure 6:**
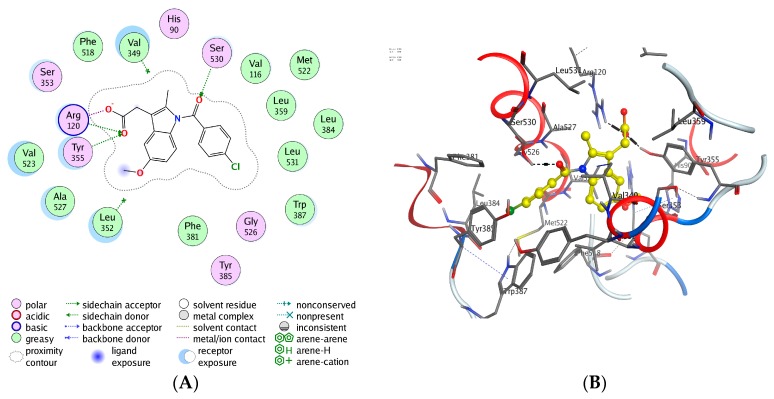
Reference compound indomethacin in the binding site of COX-2 (PDB: 4COX). (**A**,**B**) represents the 2D and 3D docking view of indomethacin binding site of COX-2. In 2D, green dashed line represents hydrogen bonding; green dashed line represents pi-H bonding; residues which are colored light green, represents hydrophobic interactions. In 3D, black dashed line represents hydrogen bonding; blue dashed line represents pi-H bonding.

Molecular docking was performed on the synthetic compounds (**S1**–**S18**) into human COX-1/COX-2 enzyme using MOE 2015 software-package and compared with the docking results of indomethacin that have been reported to have COX-2 inhibitory activity [27]. In case of docking studies of compounds to the COX-1, all the compounds fitted in the appropriate amino acids by hydrogen bonds. In addition, hydrophobic interactions were found with most of the important lipophilic amino acids: Leu 531, Leu 352, Ala 527, Ile 523, Val 349, Ser 530, Ser 353, Gly 526, and Tyr 355. From docking parameters table, we observed that the interaction of these compound form pi-H bond with energy cut-off of −0.3 kcal/mol for COX-1 and 0.5 kcal/mol for COX-2. 3D crystal structure of human COX-2 (PDB ID: 4COX) was used to interpret the differences in the binding interactions at the molecular level as inhibitors of human COX-2. (Table 5) shows the values of docking scores for compounds (**S1**–**S18**) that were docked into the site of COX-2 (PDB ID: 4COX). Docking studies have shown the potential of this series of compounds (**S1**–**S18**) to bind with COX-2. All the compounds fitted in the appropriate amino acids by hydrogen bonds. In addition, hydrophobic interactions were found with most of the important lipophilic amino acids: Leu 352, Leu 93, Tyr 355, Tyr 115, Val 116, Val 523, Val 349, Val 89, Ala 527 and Arg 120. It was found that Tyr 355 formed hydrogen bond acceptors with the carboxamide groups of all compounds except **S11**. Arg 120 also formed hydrogen bond acceptors with carboxamide groups of all compounds except **S7**, **S13**, **S17**. Leu 93 formed Pi-hydrogen bonds with the centroid of nitrophenyl of compound **S3** and trimethoxyphenyl of compound **S15.** Val 523 formed Pi-hydrogen bond with the centroid of 4-(dimethylamino) phenyl of compounds **S11** and 4-ethoxyphenyl of compound **S13**. Compound **S3** formed a hydrogen bond between OH of Tyr 355 and NH_2_ of Arg 120 with carbonyl group in compound **S3** and this hydrogen bond was similar to that formed by indomethacin. Moreover, hydrogen bond between Leu 93 with nitro-phenyl ring in compound **S3** was also formed (Figure 5, Figure 6). In 2D, green dashed line represents hydrogen bonding while; the green dashed line with ring represents pi-H bonding and the residues, which are colored light green, represents hydrophobic interactions. In 3D, black dashed line represents hydrogen bonding while; blue dashed line represents pi-H bonding. The biological activity of the compound **S3** was found to be higher than that which was expected based on data from molecular docking.

## 3. Material and Methods

### 3.1. Experimental Section

The solvents were procured from Merck. Purity of the synthesized compounds was confirmed by thin layer chromatography (TLC), performed on Silica gel 60 F_254_ coated plates (Merck, Kenilworth, NJ, USA). UV light was used for the visualization of TLC spots. Spectrum BX, Perkin Elmer FT-IR spectrophotometer (Perkin Elmer, Hopkinton, MA, USA) was used for performing FTIR. Gallenkamp melting point apparatus (Gallenkamp, Loughborough, UK) was used for performing melting points. Bruker NMR 500 MHz and 125 MHz spectrophotometer (Bruker, Billerica, MA, USA) were used for ^1^H- and ^13^C-NMR. All the samples were processed in DMSO-*d*_6_ with tetramethylsilane (TMS) as internal standard. Mass spectroscopy was used for the measurement of molecular masses of compounds. The elemental analysis of the compounds was performed on the CHN Elementar (Analysensysteme GmbH, Langenselbold, Germany). The elemental analyses were within the limit.

### 3.2. Synthesis of 2-(6-Methoxy-2-methyl-1H-indol-3-yl) Acetohydrazide *(**1**)*

The methyl ester of indomethacin (0.01 mol) and hydrazine hydrate (99%) (0.2 mol) in presence of absolute ethanol (50 mL) were refluxed for 30 h. The reaction mixture was concentrated by using rota vapor and poured in a beaker containing ice while stirring and kept for 4 h at room temperature. The solid was separated out by filtration. The product was dried and recrystallized from ethanol. The product was carefully checked by thin layer chromatography. Two compounds were isolated by column chromatography by using different fractions of n-hexane and ethyl acetate. The first compound was 2-(6-methoxy-2-methyl-1*H*-indol-3-yl) acetohydrazide compound (**1**) and was obtained as the major product. The second compound, 4-chlorobenzohydrazide (**2**) was obtained as minor product. Both the compounds were fully characterized by the spectral data.

*2-(6-methoxy-2-methyl-1H-indol-3-yl) acetohydrazide* (**1**). Color: white; Yield: 70%; m.p.: 168–170 °C; UV λmax (Methanol) = 280 nm; ^1^H-NMR (500 MHz, DMSO-*d*_6_): *δ* = 2.38 (3H, s, CH_3_), 3.54 (2H, s, CH_2_), 3.80 (3H, s, OCH_3_), 4.26 (2H, s, NH_2_, D_2_O exchg.), 6.67 (1H, d, *J* = 8.5 Hz, Ar–H), 7.16 (2H, d, *J* = 7.5 Hz, Ar–H), 9.16 (1H, s, NH, D_2_O exchg.), 10.62 (1H, s, CONH, D_2_O exchg.); ^13^C-NMR (125 MHz, DMSO-*d*_6_): *δ* = 12.0 (CH_3_), 30.2 (CH_2_), 55.8 (OCH_3_), 101.1, 105.1, 109.8, 110.0, 111.7, 128.0, 129.3, 129.7, 130.6, 134.3, 153.4, 170.8 (C=O); ms: *m*/*z* = 233.11 [M]^+^, 234.07 [M + 1]^+^; Analysis: C_12_H_15_N_3_O_2_ for, calcd. C 61.79, H 6.48, N 18.01%; found C 61.58, H 6.46, N 18.05%.

*4-Chlorobenzohydrazide* (**2**). Color: white; Yield: 20%; m.p.: 148–150 °C; UV λmax (Methanol) = 230 nm; ^1^H-NMR (500 MHz, DMSO-*d*_6_): *δ* = 4.53 (2H, s, NH_2_, D_2_O exchg.), 7.52 (2H, d, *J* = 8.5 Hz, Ar–H), 7.84 (2H, d, *J* = 8.5 Hz, Ar–H), 9.87 (1H, s, CONH, D_2_O exchg.); ^13^C-NMR (125 MHz, DMSO-*d*_6_): *δ* = 128.86, 129.32, 132.50, 136.25, 165.29; MS: *m*/*z* = 170.45 [M]^+^; Analysis: C_7_H_7_N_2_OCl for, calcd. C 49.28, H 4.14, N 16.42%; found C 49.37, H 4.12, N 16.46%.

### 3.3. General Procedure for the Synthesis of 2-(5-Methoxy-2-methyl-1-indol-3yl)-N-[(E)-Substituted Phenyl methylidine] Aceto Hydrazide Derivatives *(**S1**–**S18**)*.

A solution of indole hydrazide (**1**) (371 mg, 1.0 mmol) in EtOH (15 mL) containing an appropriate substituted benzaldehyde (1.1 mmol) and a catalytic amount of Glacial acetic acid was heated under reflux for 3 h. After cooling, 5 mL of water was added to the mixture and kept in a refrigerator for 12 h. The product was obtained by filtration. The compound was washed several times with cold water. Ethanol was used for the recrystallization of the compound.

*2-(5-Methoxy-2-methyl-1H-indol-3-yl)-N′-[(E)-phenylmethylidene] acetohydrazide* (**S1**): Yield: 70%; m.p.: 170–172 °C; IR (KBr) cm^−1^: 3412 (NH), 3024 (C–H), 1654 (C=O), 1637 (C=N); ^1^H-NMR (500 MHz, DMSO-*d*_6_): *δ =* 2.37 (3H, s, –CH_3_), 3.55 (2H, s, CH_2_), 3.74 (3H, s, –OCH_3_), 6.65–8.00 (8H, m, Ar–H), 10.62 (1H, s, =CH), 11.26 (1H, s, –NH, D_2_O exchg.), 11.9 (1H, s, CONH, D_2_O exchg.); ^13^C-NMR (125 MHz, DMSO-*d*_6_): *δ* = 12.2 (CH_3_), 28.2 (CH_2_), 55.5 (OCH_3_), 100.7, 104.7, 110.0, 111.3, 127.2, 127.4, 127.6, 129.0, 129.2, 129.3, 130.0, 134.3, 134.8, 153.3, 167.7 (C=O); MS: *m*/*z* = 321.37 [M]^+^; Analysis: for C_19_H_19_N_3_O_2_, calcd. C 71.01, H 5.96, N 13.08%; found C 71.25, H 5.94, N 13.11%.

*2-(5-Methoxy-2-methyl-1H-indol-3-yl)-N′-[(E)-(4-nitrophenyl)methylidene] acetohydrazide* (**S2**): Yield: 75%; m.p.: 220–222 °C; IR (KBr) cm^−1^: 3411 (NH), 3000 (C–H), 1654 (C=O), 1617 (C=N); ^1^H-NMR (500 MHz, DMSO-*d*_6_): *δ =* 2.37 (3H, s, –CH_3_), 3.58 (2H, s, CH_2_), 3.74 (3H, s, –OCH_3_), 6.97–8.26 (7H, m, Ar–H), 10.63 (1H, s, =CH), 11.70 (1H, s, –NH, D_2_O exchg.), 12.2 (1H, s, –CONH, D_2_O exchg.); ^13^C-NMR (125 MHz, DMSO-*d*_6_): *δ* = 11.6 (CH_3_), 28.2 (CH_2_), 55.0 (OCH_3_), 100.2, 110.8, 123.9, 124.0, 127.6, 128.0, 128.6, 129.6, 140.4, 142.0, 143.0, 144.0, 145.5, 151.0, 163.0, 175.0; MS: *m*/*z* = 366.37 [M]^+^; Analysis: for C_19_H_18_N_4_O_4_, calcd. C 62.29, H 4.95, N 15.29%; found C 62.14, H 4.97, N 15.25%.

*2-(5-Methoxy-2-methyl-1H-indol-3-yl)-N′-[(E)-(3-nitrophenyl)methylidene] acetohydrazide* (**S3**): Yield: 68%; m.p.: 200–202 °C; IR (KBr) cm^−1^: 3412 (NH), 3237 (C–H), 1654 (C=O), 1617 (C=N); ^1^H-NMR (500 MHz, DMSO-*d*_6_): *δ =* 2.37 (3H, s, –CH_3_), 3.58 (2H, s, CH_2_), 3.74 (3H, s, –OCH_3_), 6.99–8.57 (7H, m, Ar–H), 10.63 (1H, s, =CH), 11.5 (1H, s, NH, D_2_O exchg.), 12.18 (1H, s, –CONH, D_2_O exchg.); ^13^C-NMR (125 MHz, DMSO-*d*_6_): *δ* = 11.5 (CH_3_), 27.9 (CH_2_), 55.0, 100.2, 103.9, 109.4, 123.8, 124.2, 128.5, 130.3, 131.7, 133.3, 136.0, 140.33, 145.5, 148.1, 152.8, 162.2, 170.0; MS: *m*/*z* = 366.37 [M]^+^; Analysis: for C_19_H_18_N_4_O_4_, calcd. C 62.29, H 4.95, N 15.29%; found C 62.36, H 4.93, N 15.24%.

*2-(5-Methoxy-2-methyl-1H-indol-3-yl)-N′-[(E)-(2-nitrophenyl)methylidene] acetohydrazide* (**S4**): Yield: 70%; m.p.: 210–212 °C; IR (KBr) cm^−1^: 3407 (NH), 3063 (C–H), 1654 (C=O), 1617 (C=N); ^1^H-NMR (500 MHz, DMSO-*d*_6_): *δ =* 2.37 (3H, s, –CH_3_), 3.58 (2H, s, CH_2_), 3.74 (3H, s, –OCH_3_), 6.98–8.25 (7H, m, Ar–H), 10.63 (1H, s, =CH), 11.90 (1H, s, –NH, D_2_O exchg.), 12.10 (1H, s, –CONH, D2O exchg.); ^13^C-NMR (125 MHz, DMSO-*d*_6_): *δ* = 11.60 (CH_3_), 27.8 (CH_2_), 54.9 (OCH_3_), 100.0, 103.8, 109.4, 110.6, 124.5, 127.8, 129.6, 133.4, 134.0, 141.4, 143.2, 147.9, 148.2, 152.0; 167.0, 170.0; MS: *m*/*z* = 366.37 [M]^+^; Analysis: for C_19_H_18_N_4_O_4_, calcd. C 62.29, H 4.95, N 15.29%; found C 62.15, H 4.97, N 15.24%

*N′-[(E)-(4-chlorophenyl)methylidene]-2-(5-methoxy-2-methyl-1H-indol-3-yl)acetohydrazide* (**S5**): Yield: 80%; m.p.: 180–182 °C; IR (KBr) cm^−1^: 3411 (NH), 3071 (C–H), 1654 (C=O), 1597 (C=N); ^1^H-NMR (500 MHz, DMSO-*d*_6_): *δ =* 2.36 (3H, s, –CH_3_), 3.55 (2H, s, CH_2_), 3.74 (3H, s, –OCH_3_), 6.60–8.45 (7H, m, Ar–H), 10.63 (1H, s, =CH), 11.3 (1H, s, NH, D_2_O exchg.), 12.00 (1H, s, –CONH, D_2_O exchg.); ^13^C-NMR (125 MHz, DMSO-*d*_6_): *δ* = 11.6 (CH_3_), 28.2 (CH_2_), 55.1 (OCH_3_), 101.0, 128.5, 128.7, 128.8, 128.9, 129.5, 136.6, 146.7, 148.0, 149.0, 150.0, 151.0, 152.1,162.7, 172.0; MS: *m*/*z* = 355.81 [M]^+^; Analysis: for C_19_H_18_N_3_O_2_Cl, calcd. C 64.13, H 5.10, N 11.81%; found C 64.33, H 5.12, N 11.83%.

*N′-[(E)-(2,4-dichlorophenyl)methylidene]-2-(5-methoxy-2-methyl-1H-indol-3-yl)acetohydrazide* (**S6**): Yield: 65%; m.p.: 238–240 °C; IR (KBr) cm^−1^: 3411 (NH), 2940 (C–H), 1654 (C=O), 1617 (C=N); ^1^H-NMR (500 MHz, DMSO-*d*_6_): *δ =* 2.36 (3H, s, –CH_3_), 3.59 (2H, s, CH_2_), 3.74 (3H, s, –OCH_3_), 6.59–8.61 (6H, m, Ar–H), 10.62 (1H, s, =CH), 11.51 (1H, s, –NH, D_2_O exchg.); 11.7 (1H, s, –CONH, D_2_O exchg.); ^13^C-NMR (125 MHz, DMSO-*d*_6_): *δ* = 11.5 (CH_3_), 27.8 (CH_2_), 55.0 (OCH_3_), 100.2, 103.7, 109.4, 110.8, 127.8, 128.7, 129.2, 130.0, 133.4, 133.6, 134.0, 137.5, 140.9, 154.0, 168.0, 172.0S; MS: *m*/*z* = 390.26 [M]^+^; Analysis: for C_19_H_17_N_3_O_2_Cl_2_, calcd. C 58.47, H 4.35, N 10.77%; found C 58.25, H 4.33, N 10.74%.

*2-(5-Methoxy-2-methyl-1H-indol-3-yl)-N′-[(E)-(3,4-dimethoxyphenyl)methylidene] acetohydrazide* (**S7**)**:** Yield: 70%; m.p.: 210–212 °C; IR (KBr) cm^−1^: 3299 (NH), 3011 (C–H), 1654 (C=O), 1599 (C=N); ^1^H-NMR (500 MHz, DMSO-*d*_6_): *δ =* 2.40 (3H, s, –CH_3_), 3.70 (2H, s, CH_2_), 3.84 (3H, s, –OCH_3_), 6.50–8.40 (6H, m, Ar–H), 10.50 (1H, s, =CH), 11.20 (1H, s, –NH, D_2_O exchg.), 11.5 (1H, s, –CONH, D_2_O exchg.); ^13^C-NMR (125 MHz, DMSO-*d*_6_): *δ* = 12.1 (CH_3_), 12.2 (CH_2_), 55.6 (OCH_3_), 55.8 (OCH_3_), 56.0 (OCH_3_), 101.0, 121.4, 122.0, 127.5, 143.1, 151.0, 175.0; MS: *m*/*z* = 381.42 [M]^+^; Analysis: for C_21_H_23_N_3_O_4_, calcd. C 66.13, H 6.08, N 11.02%; found C 66.31, H 6.10, N 11.05%.

*2-(5-Methoxy-2-methyl-1H-indol-3-yl)-N′-[(E)-(2-methoxyphenyl)methylidene] acetohydrazide* (**S8**): Yield: 60%; m.p.: 220–222 °C; IR (KBr) cm^−1^: 3315 (NH), 3017 (C–H), 1664 (C=O), 1601 (C=N); ^1^H-NMR (500 MHz, DMSO-*d*_6_): *δ =* 2.36 (3H, s, –CH_3_), 3.56 (2H, s, CH_2_), 3.87 (3H, s, –OCH_3_), 7.00–8.82 (11H, m, Ar–H), 10.61 (1H, s, =CH), 11.23(1H, s, NH, D_2_O exchg.), 11.92 (1H, s, –CONH, D_2_O exchg.); ^13^C-NMR (125 MHz, DMSO-*d*_6_): *δ* = 12.1 (CH_3_), 28.1 (CH_2_), 56.1 (OCH_3_), 63.4 (OCH_3_), 111.2, 112.2, 112.3, 121.2, 122.7, 125.8, 126.0, 129.0, 130.0, 132.1, 137.0, 144.0, 158.2, 162.3; MS: *m*/*z* = 351.39 [M]^+^; Analysis: for C_20_H_21_N_3_O_3_, calcd. C 68.36, H 6.02, N 11.96%; found C 68.50, H 6.00, N 11.93%.

*N′-[(E)-(4-hydroxyphenyl)methylidene]-2-(5-methoxy-2-methyl-1H-indol-3-yl)acetohydrazide* (**S9**)**:** Yield: 70%; m.p.: 230–232 °C; IR (KBr) cm^−1^: 3411 (OH), 3411 (NH), 3300 (C–H), 1654 (C=O), 1609 (C=N); ^1^H-NMR (500 MHz, DMSO-*d*_6_): *δ =* 2.37 (3H, s, –CH_3_), 3.58 (2H, s, CH_2_), 3.75 (3H, s, –OCH_3_), 6.59–8.36 (7H, m, Ar–H), 9.88 (1H, s, OH, D_2_O exchg.), 10.60 (1H, s, =CH), 11.0 (1H, s, –CONH, D_2_O exchg.), 11.73 (1H, s, –CONH, D_2_O exchg.); ^13^C-NMR (125 MHz, DMSO-*d*_6_): *δ* = 12.1 (CH_3_), 28.2 (CH_2_), 55.5 (OCH_3_), 100.8, 104.9, 109.8, 110.0, 116.1, 128.9, 129.4, 130.5, 134.2, 143.4, 153.3, 159.5, 162.2, 167.4, 170.7, 172.9; MS: *m*/*z* = 337.37 [M]^+^; Analysis: for C_19_H_19_N_3_O_3_, calcd. C 67.64, H 5.68, N 12.46%; found C 67.43, H 5.70, N 12.43%.

*N′-[(E)-(3-hydroxyphenyl)methylidene]-2-(5-methoxy-2-methyl-1H-indol-3-yl)acetohydrazide* (**S10**): Yield: 60%; m.p.: 145–147 °C; IR (KBr) cm^−1^: 3500 (OH), 3413 (NH), 3023 (C–H), 1654 (C=O), 1617 (C=N); ^1^H-NMR (500 MHz, DMSO-*d*_6_): *δ =* 2.37 (3H, s, –CH_3_), 3.57 (2H, s, CH_2_), 3.74 (3H, s, –OCH_3_), 6.58–8.17 (7H, m, Ar–H), 9.59 (1H, s, OH, D_2_O exchg.), 10.62 (1H, s, =CH), 11.21 (1H, s, –NH, D_2_O exchg.), 11.39 (1H, s, –CONH, D_2_O exchg.); ^13^C NMR (125 MHz, DMSO-*d*_6_): *δ* = 11.6 (CH_3_), 27.6 (CH_2_), 54.9 (OCH_3_), 100.1, 104.0, 109.3, 110.8, 112.4, 117.2, 118.2, 128.5, 129.5, 130.0, 133.8, 135.5, 146.2, 152.8, 157.5, 175.0; MS: *m*/*z* = 337.37 [M]^+^; Analysis: for C_19_H_19_N_3_O_3_, calcd. C 67.64, H 5.68, N 12.46%; found C 67.41, H 5.70, N 12.42%.

*2-(5-Methoxy-2-methyl-1H-indol-3-yl)-N′-{(E)-[4-(dimethylamino) phenyl]methylidene}acetohydrazide* (**S11**): Yield: 65%; m.p.: 200–202 °C; IR (KBr) cm^−1^: 3351 (NH), 2909 (C–H), 1654 (C=O), 1609 (C=N); ^1^H-NMR (500 MHz, DMSO-*d*_6_): *δ =* 2.37 (3H, s, –CH_3_), 3.00 (6H, s, 2× NCH_3_) 3.59 (2H, s, CH_2_), 3.74 (3H, s, –OCH_3_), 6.59–8.32 (7H, m, Ar–H), 10.60 (1H, s, =CH), 10.97 (1H, s, –NH, D_2_O exchg.), 11.62 (1H, s, –CONH, D_2_O exchg.); ^13^C-NMR (125 MHz, DMSO-*d*_6_): *δ* = 12.1 (CH_3_), 28.0 (CH_2_), 30.0 (NCH_3_), 31.0 (NCH_3_), 55.5 (OCH_3_), 100.9, 105.0, 110.0, 111.2, 112.27, 112.3, 122.2, 128.4, 128.7, 128.9, 129.8, 130.5, 143.9, 151.7, 153.3, 172.7; ms: *m*/*z* = 364.44 [M]^+^; Analysis: for C_21_H_24_N_4_O_2_, calcd. C 69.21, H 6.64, N 15.37%; found C 69.37, H 6.66, N 15.33%.

*2-(5-methoxy-2-methyl-1H-indol-3-yl)-N′-[(E)-(3-methoxyphenyl)methylidene] acetohydrazide* (**S12**): Yield: 65%; m.p.: 195–197 °C; IR (KBr) cm^−1^: 3412 (NH), 3000 (C–H), 1654 (C=O), 1636 (C=N); ^1^H-NMR (500 MHz, DMSO-*d*_6_): *δ =* 2.37 (3H, s, –CH_3_), 3.58 (2H, s, CH_2_), 3.80 (3H, s, –OCH_3_), 6.59–8.44 (7H, m, Ar–H), 10.67 (1H, s, =CH), 11.28 (1H, s, –NH, D_2_O exchg.), 11.46 (1H, s, –CONH, D_2_O exchg.); ^13^C-NMR (125 MHz, DMSO-*d*_6_): *δ* = 12.1 (CH_3_), 28.3 (CH_2_), 55.5 (OCH_3_), 55.35 (OCH_3_), 100.8, 104.5, 104.7, 109.8, 110.0, 111.6, 120.3, 129.3, 130.3, 134.3, 134.3, 136.2, 142.9, 146.5, 153.3, 159.9, 173.3; MS: *m*/*z* = 351.39 [M]^+^; Analysis: for C_20_H_21_N_3_O_3_, calcd. C 68.36, H 6.02, N 11.96%; found C 68.15, H 6.00, N 11.99%.

*N′-[(E)-(4-ethoxyphenyl)methylidene]-2-(5-methoxy-2-methyl-1H-indol-3-yl)acetohydrazide* (**S13**): Yield: 75%; m.p.: 213–215 °C; IR (KBr) cm^−1^: 3322 (NH), 3042 (C–H), 1654 (C=O), 1571 (C=N); ^1^H-NMR (500 MHz, DMSO-*d*_6_): *δ =* 1.33 (3H, t, *J* = 7.5 Hz, CH_2_CH_3_), 2.37 (3H, s, –CH_3_), 3.57 (2H, s, CH_2_), 3.74 (3H, s, –OCH_3_), 4.06 (2H, q, *J* = 7.5 Hz, OCH_2_), 6.58–8.20 (7H, m, Ar–H), 10.61 (1H, s, =CH), 11.12 (1H, s, –NH, D_2_O exchg.), 11.30 (1H, s, –CONH, D_2_O exchg.); ^13^C-NMR (125 MHz, DMSO-*d*_6_): *δ* = 12.2 (CH_3_), 15.0 (CH_2_), 28.2 (CH_3_), 55.5 (OCH_3_), 55.88 (OCH_2_), 63.7, 100.8, 104.8, 109.8, 111.2, 115.1, 127.2, 129.2, 129.3, 130.5, 134.3, 143.0, 146.5, 153.3, 160.2, 167.5, 173.0; ms: *m*/*z* = 365.42 [M]^+^; Analysis: for C_21_H_23_N_3_O_3_, calcd. C 69.02, H 6.34, N 11.50%; found C 69.22, H 6.36, N 11.53%.

*2-(5-Methoxy-2-methyl-1H-indol-3-yl)-N′-[(E)-(2,4,5-trimethoxyphenyl) methylidene]acetohydrazide* (**S14**): Yield: 60%; m.p.: 238–240 °C; IR (KBr) cm^−1^: 3412 (NH), 2943 (C–H), 1654 (C=O), 1617 (C=N); ^1^H-NMR (500 MHz, DMSO-*d*_6_): *δ =* 2.36 (3H, s, –CH_3_), 3.51 (2H, s, CH_2_), 3.78 (12H, s, 4× –OCH_3_), 6.91–8.42 (5H, m, Ar–H), 10.61 (1H, s, =CH), 11.14 (1H, s, –NH, D_2_O exchg.), 11.42 (1H, s, –CONH, D_2_O exchg.); ^13^C-NMR (125 MHz, DMSO-*d*_6_): *δ* = 12.1 (CH_3_), 30.1 (CH_2_), 55.5 (OCH_3_), 56.4 (OCH_3_), 60.9 (OCH_3_), 62.1 (OCH_3_), 100.8, 104.8, 109.1, 109.8, 110.0, 111.2, 120.8, 130.5, 134.4, 138.9, 142.0, 152.8, 153.3, 155.2, 172.9; ms: *m*/*z* = 411.45 [M]^+^; Analysis: for C_22_H_25_N_3_O_5_, calcd. C 64.22, H 6.12, N 10.21%; found C 64.35, H 6.14, N 10.24%.

*2-(5-Methoxy-2-methyl-1H-indol-3-yl)-N′-[(E)-(2,3,4-trimethoxyphenyl) methylidene]acetohydrazide* (**S15**): Yield: 55%; m.p.: 250–252 °C; IR (KBr) cm^−1^: 3310 (NH), 3048 (C–H), 1654 (C=O), 1595 (C=N); ^1^H-NMR (500 MHz, DMSO-*d*_6_): *δ =* 2.37 (3H, s, –CH_3_), 3.59 (2H, s, CH_2_), 3.84 (12H, s, 4× –OCH_3_), 6.59–8.74 (5H, m, Ar–H), 10.60 (1H, s, =CH), 11.08 (1H, s, –NH, D_2_O exchg.), 11.33 (1H, s, –CONH, D_2_O exchg.); ^13^C-NMR (125 MHz, DMSO-*d*_6_): *δ* = 12.1 (CH_3_), 28.5 (CH_2_), 55.6 (OCH_3_), 56.2 (OCH_3_), 56.4 (OCH_3_), 56.9 (OCH_3_), 98.3, 101.1, 104.8, 108.4, 109.8, 111.2, 114.1, 129.2, 130.6, 134.4, 138.8, 143.6, 152.1, 153.6, 167.3, 172.9; ms: *m*/*z* = 411.45 [M]^+^; Analysis: for C_22_H_25_N_3_O_5_, calcd. C 64.22, H 6.12, N 10.21%; found C 64.36, H 6.10, N 10.23%.

*2-(5-Methoxy-2-methyl-1H-indol-3-yl)-N′-[(E)-(3,4,5-trimethoxyphenyl) methylidene]acetohydrazide* (**S16**): Yield: 58%; m.p.: 233–235 °C; IR (KBr) cm^−1^: 3309 (NH), 3015 (C–H), 1654 (C=O), 1577 (C=N); ^1^H-NMR (500 MHz, DMSO-*d*_6_): *δ =* 2.37 (3H, s, –CH_3_), 3.59 (2H, s, CH_2_), 3.83 (12H, s, 4× –OCH_3_), 6.97–8.20 (5H, m, Ar–H), 10.61 (1H, s, =CH), 11.28 (1H, s, –NH, D_2_O exchg.), 11.40 (1H, s, –CONH, D_2_O exchg.); ^13^C-NMR (125 MHz, DMSO-*d*_6_): *δ* = 12.1 (CH_3_), 28.4 (CH_2_), 55.6 (OCH_3_), 55.8 (OCH_3_), 56.3 (OCH_3_), 60.5 (OCH_3_), 60.5, 101.1, 104.4, 104.6, 104.7, 109.8, 111.2, 130.3, 130.6, 134.2, 139.3, 142.9, 153.3, 153.6, 173.2; ms: *m*/*z* = 411.45 [M]+; Analysis: for C_22_H_25_N_3_O_5_, calcd. C 64.22, H 6.12, N 10.21%; found C 64.37, H 6.10, N 10.24%.

*2-(5-Methoxy-2-methyl-1H-indol-3-yl)-N′-[(E)-(2,4,6-trimethoxyphenyl) methylidene]acetohydrazide* (**S17**): Yield: 55%; m.p.: 230–232 °C; IR (KBr) cm^−1^: 3412 (NH), 3056 (C–H), 1654 (C=O), 1612 (C=N); ^1^H-NMR (500 MHz, DMSO-*d*_6_): *δ =* 2.37 (3H, s, –CH_3_), 3.59 (2H, s, CH_2_), 3.82 (12H, s, 4× –OCH_3_), 6.57–8.74 (5H, m, Ar–H), 10.61 (1H, s, =CH), 11.10 (1H, s, NH, D_2_O exchg.), 11.80 (1H, s, –CONH, D_2_O exchg.); ^13^C NMR (125 MHz, DMSO-*d*_6_): *δ* = 12.1 (CH_3_), 28.4 (CH_2_), 55.5 (OCH_3_), 55.7 (OCH_3_), 56.3 (OCH_3_), 60.5 (OCH_3_), 98.6, 106.7, 111.2, 115.6, 129.9, 142.2, 144.1, 153.3, 159.3, 159.6 162.1, 167.3, 172.9; ms: *m*/*z* = 411.45 [M]+; Analysis: for C_22_H_25_N_3_O_5_, calcd. C 64.22, H 6.12, N 10.21%; found C 64.38, H 6.13, N 10.17%.

*2-(5-Methoxy-2-methyl-1H-indol-3-yl)-N′-[(E)-(2,4-dimethoxyphenyl) methylidene]acetohydrazide* (**S18**): Yield: 60%; m.p.: 170–172 °C; IR (KBr) cm^−1^: 3413 (NH), 3000 (C–H), 1654 (C=O), 1638 (C=N); ^1^H-NMR (500 MHz, DMSO-*d*_6_): *δ =* 2.40 (3H, s, –CH_3_), 3.60 (2H, s, CH_2_), 3.82 (3H, s, –OCH_3_), 6.50–8.70 (6H, m, Ar–H), 10.61 (1H, s, =CH), 11.20 (1H, s, NH, D_2_O exchg.); 11.80 (1H, s, –CONH, D_2_O exchg.); ^13^C-NMR (125 MHz, DMSO-*d*_6_): *δ* = 11.5 (CH_3_), 28.4 (CH_2_), 55.0 (OCH_3_), 55.6 (OCH_3_), 55.68 (OCH_3_), 98.2, 104.4, 106.2, 109.5, 110.7, 115.2, 128.8, 130.0, 132.0, 135.8, 138.3, 143.6, 152.8, 158.9, 162.0, 162.4, 164.7, 172.0; ms: *m*/*z* = 381.42 [M]^+^; Analysis: for C_21_H_23_N_3_O_4_, calcd. C 66.13, H 6.08, N 11.02%; found C 66.34, H 6.10, N 11.05%.

### 3.4. Anti-Inflammatory Activity

Anti-inflammatory activity was evaluated by reported carrageenan-induced rat paw edema method. Male rats weighing 200 g were housed in a room with a controlled temperature and 12 h light/12 h dark cycle. Each group consisted of six randomly assigned rats. Compounds were administered p.o. as suspension prepared in 1% methyl cellulose. Intradermal injection of 50 µL of 1% carrageenan induced into the sub plantar region of the right hind paw was used for paw edema, after one hour of test compound administration. Activity was evaluated after p.o. administration of indomethacin or test compounds at the dose of (10 mg/kg) each. Immediately after dosing and after 2 h and 3 h, paw volume was measured by using a plethysmometer (UGO 7140 plethysmometer). The control group was given vehicle (1% methyl cellulose) only. Percent inhibition was calculated taking the values in the control group as 0% inhibition.

### 3.5. Analgesic Activity

Male albino Swiss mice (25 g) used in the study were distributed into various groups (*n* = 6). Each mouse was initially placed on a hot plate (hot plate analgesia meter, Harvard apparatus Ltd.) thermostatically maintained at 58 °C. The mouse was monitored carefully for the time in seconds in which it displayed nociceptive responses, considered as the control reaction time. To avoid damage to the paws, a cut off time of 60 s was used. Activity was evaluated after p.o. administration of indomethacin or test compounds at the dose of (20 mg/kg) each. The reaction time was then retested at 0 h and 120 min after injection (each animal acted as its own control). The percentage changes in the reaction were then calculated.

### 3.6. Ulcerogenic Activity

For acute ulcerogenesis testing, albino rats, six rats in each group were used (Experimental animal ethical approval number: 7570). Activity was evaluated after p.o. administration of indomethacin or test compounds at the dose of (20 mg/kg) each. All of the test compounds were given as equimolar oral doses. Suspension of 1% methyl cellulose p.o. was given to control group. The stomach was opened along the greater curvature, washed with distilled water. Magnifying glass was used to examine the mucosal damage. For each stomach, the mucosal damage was assessed according to the reported scoring system.

### 3.7. Lipid Peroxidation

For determination of lipid peroxidation, the gastric mucosa was scraped with two glass slides, weighed (100 mg) and homogenized in 1.8 mL of 1.15% ice cold KCl solution. The reported procedure was followed for processing the samples. The supernatant organic layer was separated out and absorbance was measured by UV spectrophotometer at 532 nm. The results were expressed as nmol MDA/100 mg tissue.

### 3.8. Ethanol Induced Ulcer Model

The ethanol induced ulcer model was used to study gastro-protective activity of compound **S3**. The rats were grouped into five groups (*n* = 6). Group I and II received saline solution and served as negative-control and ulcer-control, respectively. Group III received compound **S3** (25.6 mg/kg) orally and served as the experimental drug group. Rats in groups IV received the indomethacin (25 mg/kg bodyweight). All of the groups received (20 mL/kg) of ethanol 95% except group I. One h alter, the rats were sacrificed under anesthesia and their stomachs were removed for further experimental studies.

Periodic Acid-Schiff (PAS) staining was used to detect the glycogen level in control and pretreated rats. With regard to the gross gastric lesion evaluation, the stomach of each rat was opened along the greater arc and washed with saline water to remove gastric contents. Gastric ulcers appear as elongated bands on the gastric mucosa. A digital planimeter was used to measure the ulcers area (hemorrhagic lesions). The length and width of each lesion were measured in (mm^2^). To determine mucus production of the gastric mucosa, the gastric mucosa of each rat was obtained and weighed using a high precision electronic balance. For the histologic studies, gastric tissues were fixed in 4% formalin solution after sacrifice. Then, each tissue sample was embedded in paraffin and cut into 5 μm thick slices for histopathological evaluation. Hematoxylin and eosin (H & E) were used for tissue section staining.

### 3.9. Determination of LD_50_

The LD_50_ (lethal dose 50%) of compound **S3** was calculated by Karber method. For determination of LD_50_, an observation was made for 24 h and symptoms of toxicity and rate of mortality were noted. Expired rats were counted at the end of the study period for the calculation of LD_50_. LD_50_ = LD_100_ − ∑ × (a × b)/*n*, where “*n*” is the total number of rats in a group, “a” is the difference between two successive doses of administered substance, “b” is the average number of dead rats in two successive doses, and LD_100_ is the lethal dose causing 100% death of all tested rats.

### 3.10. Western Blot

For the Western blot analysis, 20 µg of protein was shifted to PVDF membranes, restricted in 5% skim milk in TBS buffer 1% Tween 20, and then incubated overnight with the COX-1, COX-2 and β-actin, followed by HRP-conjugated anti-rat/rabbit/goat antibodies for 2 h at 25 °C. Bands were visualized with the Luminata Western Chemiluminescent HRP Substrates and densitometry analysis of bands was assessed using LI-COR C-DiGit blot scanners.

### 3.11. Docking Studies of Compounds

Docking for the synthesized compounds (**S1**–**S18**) and reference compound indomethacin was performed via their 3D structures and energy was minimized using MMFF94x of Molecular Operating Environment energy minimization module (MOE, Version 2015, Chemical Computing Group Inc., Montreal, Quebec, Canada).3D crystal structure of COX-2 enzyme (PDB ID: 4COX) was selected from Protein Data Bank database (http://www.rcsb.org). The reported protocol was followed for preparing selected 3D structure of COX-2 enzyme for docking. Docking procedure was also followed using the standard protocol implemented in MOE 2015 and the geometry of resulting complexes was studied using the MOE’s Pose Viewer utility.

## 4. Conclusions

In conclusion, novel indole hydrazide derivatives were synthesized in good yield. All the compounds were fully characterized by spectral data and elemental analysis. Applying the chemical modifications to the indole hydrazide scaffold resulted in eighteen derivatives with significant anti-inflammatory activity. Three compounds namely **S3**, **S7** and **S14** exhibited considerable anti-inflammatory activities compared to the reference drug indomethacin. These compounds were found to be highly significant anti-inflammatory and analgesic agents in the order of **S14** > **S3** > **S7**. The compounds were less ulcerogenic than the reference drug indomethacin in the order of **S3** > **S7** > **S14**. Compound **S3** (R = 3-nitro phenyl) was found to be a potent anti-inflammatory and analgesic agent with significant gastric sparing activity. Lead compound **S3**, presented maximum reduction in gastric ulceration and lipid peroxidation. The toxicity of compound **S3** was found to be very low; with significant gastro-protective activity. It was observed that 3-nitrophenyl substitution to the indole hydrazide has significant effect on the anti-inflammatory, analgesic and ulcerogenic reduction activities. It was found to have all the potential specification as a lead compound, which was subjected for further biological assay. It was also found to be a potent inhibitor of the COX-2 expression. Docking studies have shown the potential of this series of compounds (**S1**–**S18**) to bind with COX-2. It formed a hydrogen bond between OH of Tyr 355 and NH2 of Arg 120 with carbonyl group and this hydrogen bond was similar to that formed by indomethacin. These results provide the essential information for developing new anti-inflammatory and analgesic agent with gastro-protective effect.

## Figures and Tables

**Table 1:** Anti-inflammatory activity of compounds (**S1**–**S18**).

Treatments	Increase in Paw Volume (mm)		% Inhibition		Potency
	After 2 h	After 3 h	After 2 h	After 3 h	
**S1**	0.53 ± 0.04	0.53 ± 0.04 ***	43.95	44.31	0.57
**S2**	0.81 ± 0.03	0.79 ± 0.02 ***	14.71	17.93	0.21
**S3**	0.36 ± 0.04 ***	0.37 ±0.04 ***	61.99	61.20	0.79
**S4**	0.43 ± 0.01 ***	0.43 ± 0.02 ***	54.46	55	0.71
**S5**	0.82 ± 0.07	0.85 ± 0.06	13.48	11.89	0.16
**S6**	0.46 ± 0.04 ***	0.46 ± 0.05 ***	51.31	51.89	0.66
**S7**	0.36 ± 0.02 ***	0.36 ± 02 ***	61.47	62.24	0.80
**S8**	0.91 ± 0.04	0.95 ± 0.03	4.20	0	0.02
**S9**	0.57 ± 0.02 ***	0.51 ± 0.03 ***	39.40	46.37	0.55
**S10**	0.68 ± 0.02 ***	0.69 ± 0.01 ***	28.19	27.75	0.36
**S11**	0.45 ± 0.02 ***	0.43 ± 0.03 ***	52.18	55	0.69
**S12**	0.81 ± 0.03 *	0.79 ± 0.03 **	14.71	18.27	0.21
**S13**	0.76 ± 0.33 **	0.82 ± 0.04 *	19.96	14.65	0.22
**S14**	0.35 ± 0.02 ***	0.34 ± 0.02 ***	62.69	63.69	0.82
**S15**	0.88 ± 0.01	0.89 ± 0.09	7.35	7.06	0.09
**S16**	0.78 ± 0.04 *	0.79 ± 0.04 *	17.68	17.41	0.22
**S17**	0.49 ± 0.02 ***	0.48 ± 0.02 ***	47.81	50.34	0.63
**S18**	0.69 ± 0.12	0.62 ± 0.05 ***	27.49	35.68	0.40
**indomethacin**	0.20 ± 0.02 ***	0.22 ± 0.02 ***	77.23	76.89	1.00
**Control**	0.95 ± 0.02	0.96 ± 0.02	-	-	-

All values represent mean ± SEM.* *p* < 0.05, ** *p* < 0.01, *** *p* < 0.001; ANOVA, followed by Dunnett’s multiple comparison test (*n* = 6) All data were compare with control group.

**Table 2:** Analgesic activities of selected compounds.

Treatments	Pretreatment (0 h)	Post Treatment (3 h)	% Inhibition	Potency
	Mean ± SE (Second)	Mean ± SE (Second)		
**S1**	8.33 ± 0.49	10.83 ± 0.79 *	30	0.35
**S3**	7.33 ± 0.42	11.83 ± 0.65 ***	61.36	0.72
**S4**	6.83 ± 0.30	7.33 ± 0.40	7.31	0.08
**S6**	7.33 ± 0.33	8.33 ± 0.49	13.63	0.16
**S7**	7.33 ± 0.42	10.83 ± 0.47 ***	47.72	0.56
**S9**	7.16 ± 0.30	11.16 ± 0.60 ***	55.81	0.66
**S10**	6.66 ± 0.33	10.83 ± 0.47 ***	62.50	0.74
**S11**	7.16 ± 0.47	10.16 ± 0.65 **	41.86	0.49
**S14**	6.16 ± 0.30	10.50 ± 0.42 ***	70.27	0.83
**S17**	6.50 ± 0.22	10.50 ± 0.42 ***	61.53	0.73
**S18**	6.16 ± 0.30	7.66 ± 0.33 **	24.32	0.28
**indomethacin**	7.33 ± 0.42	13.50 ± 0.42 ***	84.09	1.00

All values represent mean ± SEM.* *p* < 0.05, ** *p* < 0.01, *** *p* < 0.001; ANOVA, followed by Dunnett’s multiple comparison test, (*n* = 6). All data were compared with control group.

**Table 3:** Ulcerogenic and lipid peroxidation activity of compounds (**S1**–**S18**) in liver and kidney tissue.

Treatments	Ulcerogenic Activity (Index)		Nanomoles of MDA Content (Liver tissue)		Nanomoles of MDA Content (Kidney Tissue)	
	Mean ± SE	% Inhibition	Mean ± SEM/100 mg Tissue	% Change	Mean ± SEM/100 mg Tissue	% Change
**S1**	0.582 ± 0.17	38.6	7.00 ± 0.25 *	14.13	4.78 ± 0.14 ***	28.66
**S2**	0.362 ± 0.17	61.81	5.94 ± 0.25 ***	27.22	5.89 ±0.14 **	12.10
**S3**	0.116 ± 0.07 **	87.76	5.55 ± 0.18 ***	31.93	6.83 ± 0.17	1.91
**S4**	0.00	100	4.70 ± 5.75 ***	40.32	4.01 ± 0.14 ***	40.12
**S5**	0.532 ± 0.08	43.88	6.08 ± 0.18 ***	18.84	4.40 ± 0.27 ***	40.12
**S6**	0.00	100	4.35 ± 0.16 ***	45.59	3.80 ± 0.12 ***	43.31
**S7**	0.432 ± 0.04 *	54.30	6.32 ± 0.14 ***	22.51	5.64 ± 0.20 **	15.92
**S8**	0.132 ± 0.08 **	86.07	6.88 ± 0.37 *	15.70	5.94 ± 0.18 *	11.46
**S9**	0.064 ± 0.03 **	93.24	7.82 ± 0.28	4.18	6.02 ± 0.19 *	10.19
**S10**	0.948 ± 0.17	0	8.58 ± 0.38	5.23	7.17 ± 0.20	7.00
**S11**	0.00	100	4.74 ±0.28 ***	41.88	4.14 ± 0.18 ***	38.21
**S12**	0.316 ± 0.09 *	66.66	5.85 ± 0.18 ***	28.27	5.29 ± 0.28 **	21.09
**S13**	0.696 ± 0.09	26.58	5.29 ± 0.17 ***	35.07	5.12 ± 0.16 ***	23.56
**S14**	0.616 ± 0.11	35.2	5.59 ± 0.19 ***	41.36	5.68 ± 0.12 **	15.28
**S15**	0.00	100	4.78 ± 0.06 ***	41.36	4.01 ± 0.23 ***	40.12
**S16**	0.966 ± 0.16	0	7.56 ± 0.43	7.32	7.17 ± 0.22	7.00
**S17**	0.00	100	4.65 ± 0.21 ***	42.93	4.44 ± 0.17 ***	33.75
**S18**	0.598 ± 0.11	36.91	5.81 ± 0.41 ***	28.79	5.64 ± 0.18 **	15.92
**indomethacin**	0.948 ± 0.21		8.16 ± 0.28 ***	114.60	6.70 ± 0.20 ***	98.73
**Control**	0.00	100	3.80 ± 0.18	-	3.37 ± 0.12	

Values represent mean ± SEM.* *p* < 0.05, ** *p* < 0.01, *** *p* < 0.001; ANOVA, followed by Dunnett’s multiple comparison test (*n* = 6). All data were compared with Indomethacin group.

**Table 4:** Ulcer index of compound **S3** compared with standard drug indomethacin.

Animal Groups	Treatment (5 mL/kg Dose)	Ulcer Index (mm^2^)
		Mean ± SEM
1	**normal control**	0
2	**ethanol group**	7.83 ± 0.33
3	**S3 (25.6 mg/kg)**	2.83 ± 0.87
4	**Indomethacin (25 mg/kg)**	12.34 ± 0.73

**Table 5:** Docking of compounds (**S1**–**S18**) into COX-2 with reference drug indomethacin.

Compd. No.	Amino Acid Residues	Interaction Type	Distance (Å)	Total Binding Energy (kcal·mol^−1^)	RMSD
**indomethacin**	SER 530	H-acceptor	2.92	−8.86	0.748
	ARG 120	H-acceptor	2.84		
	TYR 355	H-acceptor	2.84		
	ARG 120	ionic	2.42		
	ARG 120	ionic	3.04		
**S1**	ARG 120	H-acceptor	2.87	−7.12	2.008
	TYR 355	H-acceptor	2.87		
**S2**	ARG 120	H-acceptor	2.78	−7.73	2.008
	TYR 355	H-acceptor	3.04		
**S3**	ARG 120	H-acceptor	2.80	−7.80	2.0
	TYR 355	H-acceptor	3.08		
	LEU 93	Pi-H	4.46		
**S4**	ARG 120	H-acceptor	2.82	−7.86	1.322
	TYR 355	H-acceptor	2.97		
**S5**	ARG 120	H-acceptor	2.87	−7.79	1.480
	TYR 355	H-acceptor	2.88		
**S6**	ARG 120	H-acceptor	2.83	−7.80	1.645
	TYR 355	H-acceptor	2.94		
**S7**	TYR 355	H-acceptor	2.75	−7.47	1.058
**S8**	ARG 120	H-acceptor	2.88	−7.997	1.365
	TYR 355	H-acceptor	3.06		
**S9**	ARG 120	H-acceptor	2.88	−7.31	1.606
	TYR 355	H-acceptor	2.99		
**S10**	ARG 120	H-acceptor	2.85	−7.52	1.851
	TYR 355	H-acceptor	2.96		
**S11**	ARG 120	H-acceptor	2.79	−7.16	0.941
	VAL 523	pi-H	4.61		
**S12**	ARG 120	H-acceptor	2.87	−7.73	2.050
	TYR 355	H-acceptor	2.87		
**S13**	TYR 355	pi-H	3.46	−7.48	1.474
	VAL 523	pi-H	4.77		
**S14**	ARG 120	H-acceptor	2.83	−8.38	1.357
	TYR 355	H-acceptor	2.92		
**S15**	ARG 120	H-acceptor	2.87	−8.49	2.301
	TYR 355	H-acceptor	2.87		
	LEU 93	pi-H	4.56		
**S16**	ARG 120	H-acceptor	2.82	−8.22	2.279
	TYR 355	H-acceptor	2.90		
**S17**	TYR 355	H-acceptor	3.12	−7.71	2.563
**S18**	ARG 120	H-acceptor	3.35	−7.60	1.363

## Supplementary Materials

The following are available online.
