# Supplementary material for: Indole Derivatives as Cyclooxygenase Inhibitors: Synthesis, Biological Evaluation and Docking Studies
Source: Molecules. 2018 May 24;23(6):1250. doi: 10.3390/molecules23061250 (PMC6100013; doi:10.3390/molecules23061250)
Supplement: Supplementary file 1 [file molecules-23-01250-s001.pdf]

Table: Comparison molecular docking parameters obtained from the selected compounds binding to COX-1 and COX-2.

Compd. NO.	COX-2 (4COX)				COX-1 (3KK6)			
	Amino Acid Residues	Interaction Type	Distance (Å)	Total Binding Energy (kcal·mol⁻¹)	Amino Acid Residues	Interaction Type	Distance (Å)	Total Binding Energy (kcal·mol⁻¹)
**IMN CEL**	SER 530<br>ARG 120<br>TYR 355<br>ARG 120<br>ARG 120	H-acceptor<br>H-acceptor<br>H-acceptor<br>ionic<br>ionic	2.92<br>2.84<br>2.84<br>2.42<br>3.04	-8.86	GLN 192<br>LEU 352<br>ILE 517<br>HIS 90	H-donor<br>H-donor<br>H-acceptor<br>H-acceptor	2.33<br>2.23<br>3.45<br>3.32	8.51
**S1**	ARG 120<br>TYR 355	H-acceptor<br>H-acceptor	2.87<br>2.87	-7.12	TYR 355<br>ALA 527	H-acceptor<br>pi-H	2.87<br>3.40	-7.71
**S2**	ARG 120<br>TYR 355	H-acceptor<br>H-acceptor	2.78<br>3.04	-7.73	ALA 527	pi-H	3.87	-8.25
**S3**	ARG 120<br>TYR 355<br>LEU 93	H-acceptor<br>H-acceptor<br>Pi-H	2.80<br>3.08<br>4.46	-7.80	TYR 355<br>ALA 527	H-acceptor<br>pi-H	2.95<br>3.35	-8.15
**S4**	ARG 120<br>TYR 355	H-acceptor<br>H-acceptor	2.82<br>2.97	-7.86	TYR 355<br>ILE 523<br>ILE 523	H-acceptor<br>H-acceptor<br>pi-H	3.21<br>2.69<br>3.83	-7.67
**S5**	ARG 120<br>TYR 355	H-acceptor<br>H-acceptor	2.87<br>2.88	-7.79	LEU 531	pi-H	4.38	-7.11
**S6**	ARG 120<br>TYR 355	H-acceptor<br>H-acceptor	2.83<br>2.94	-7.80	PHE 518<br>ALA 527	H-acceptor<br>pi-H	3.44<br>3.88	-7.42

**S7**	TYR 355	H-acceptor	2.75	-7.47	SER 353<br>ILE 523	pi-H<br>pi-H	3.46<br>3.88	-7.47
**S8**	ARG 120<br>TYR 355	H-acceptor<br>H-acceptor	2.88<br>3.06	-7.997	ALA 527	pi-H	3.43	-7.21
**S9**	ARG 120<br>TYR 355	H-acceptor<br>H-acceptor	2.88<br>2.99	-7.31	ILE 523<br>ALA 527	H-acceptor<br>pi-H	3.29<br>4.14	-7.78
**S10**	ARG 120<br>TYR 355	H-acceptor<br>H-acceptor	2.85<br>2.96	-7.52	-	-		-7.79
**S11**	ARG 120<br>VAL 523	H-acceptor<br>pi-H	2.79<br>4.61	-7.16	ALA 527	pi-H	3.51	-6.92
**S12**	ARG 120<br>TYR 355	H-acceptor<br>H-acceptor	2.87<br>2.87	-7.73	-	-	-	-7.94
**S13**	TYR 355<br>VAL 523	pi-H<br>pi-H	3.46<br>4.77	-7.48	-	-	-	-8.21
**S14**	ARG 120<br>TYR 355	H-acceptor<br>H-acceptor	2.83<br>2.92	-8.38	TYR 355	pi-H	3.39	-6.31
**S15**	ARG 120<br>TYR 355<br>LEU 93	H-acceptor<br>H-acceptor<br>pi-H	2.87<br>2.87<br>4.56	-8.49	TYR 355	H-acceptor	2.91	-5.63
**S16**	ARG 120<br>TYR 355	H-acceptor<br>H-acceptor	2.82<br>2.90	-8.22	-	-	-	-6.52
**S17**	TYR 355	H-acceptor	3.12	-7.71	ILE 523	H-acceptor	3.11	-7.76
**S18**	ARG 120	H-acceptor	3.35	-7.60	TYR 355<br>ALA 527	H-acceptor<br>pi-H	2.99<br>3.50	-7.25
